# Conservative Treatment of Stage IA1 Adenocarcinoma of the Uterine Cervix during Pregnancy: Case Report and Review of the Literature

**DOI:** 10.1155/2014/296253

**Published:** 2014-03-16

**Authors:** Francesco Sopracordevole, Diego Rossi, Jacopo Di Giuseppe, Marta Angelini, Pierino Boschian-Bailo, Monica Buttignol, Andrea Ciavattini

**Affiliations:** ^1^Department of Gynecologic Oncology, Centro di Riferimento Oncologico-National Cancer Institute, 33081 Aviano, Pordenone, Italy; ^2^Department of Pathology, Centro di Riferimento Oncologico-National Cancer Institute, 33081 Aviano, Pordenone, Italy; ^3^Obstetric and Gynecologic Clinic, University of Udine, 33100 Udine, Italy; ^4^Obstetric and Gynecologic Unit, Palmanova General Hospital, 33057 Palmanova, Italy; ^5^Woman's Health Sciences Department, Polytechnic University of Marche, 60123 Ancona, Italy

## Abstract

Microinvasive adenocarcinoma (MIAC) of the uterine cervix is rare in pregnancy. Published data on conservative treatment of MIAC both in pregnant and nonpregnant women are scarce. A conservatively treated case of MIAC in a 13-week-pregnant woman after a diagnosis of atypical glandular cells (AGC) on pap smear at the 6th week of pregnancy is presented. The problems of suspected adenocarcinoma in situ (AIS) on biopsy and MIAC on cone biopsy in pregnancy, as well as the risks and benefits of a conservative treatment are discussed. After colposcopic guide laser cervical conization and expression of informed consent the patient underwent followup and vaginal delivery at 40 weeks plus 3 days of gestation. In this case, no obstetric complication has been recorded after the cervical conization, and after a followup of 18 months the patient was alive and free of disease, with negative results as far as pap smear, colposcopy, HPV status, and cervical curettage are concerned. In a stage Ia1 disease of endocervical type, with clear margins and without lymph-vascular space invasion, cervical conization performed during the second trimester may be considered a definitive and safe treatment, at least up to delivery, after expression of informed consent by the woman.

## 1. Introduction

Microinvasive adenocarcinoma of the cervix (MIAC) is occasionally found in the definitive histology in the specimens of conization performed because of squamous or glandular intraepithelial cervical neoplasia or suspicious for adenocarcinoma in situ (AIS). MIAC corresponds to FIGO stages Ia1 and Ia2, and its frequency, compared to all microinvasive cervical cancers (MICC), is about 12% [1]. The large presence of cytological-based screening programs in developed countries led to an increasing number of diagnoses in younger women [2], often in childbearing age. There are enough data on the safety of fertility-sparing treatment of squamous microinvasive cancers (MISC) [3–5] but few on fertility sparing treatment of MIAC [6, 7], in particular, with long-term followup [8, 9]. The occurrence of cervical cancer during pregnancy involves ethical and technical problems, related to the survival of the woman and fetal viability. Although the conservative treatment of MISC [10, 11] and small stage IB1 squamous cancer during pregnancy [12, 13] seems to be safe, scarce data are available for MIAC conservative treatment in pregnancy [2]. This case report is an addition to the few published cases of conservatively treated MIACs during pregnancy.

## 2. Case Presentation

A 32-year-old pregnant woman, PARA 0000, underwent pap smear at the 6th week of pregnancy, 40 months after her latest negative test; the diagnosis was atypical glandular cells—not otherwise specified (AGC-nos). At the age of 22 she had regular screening pap smear tests every three years, with negative results for intraepithelial or invasive cervical lesions and abnormal glandular cells. She did not smoke, and the HIV test at the beginning of the pregnancy was negative. She had taken contraceptive pills for five years, until the age of 30; nothing else was obtained from her gynaecological history.

A colposcopy was performed 15 days later; it was satisfactory, with squamous-columnar junction (SCJ) fully visible, and showed grade 2 abnormal transformation zone because of the extension of thick acetowhite iodine-negative epithelium towards the cervical canal and partial covering of the glandular epithelium; atypical vessels were found inside the glandular epithelium, close to the SCJ, both in the anterior and the posterior lips of the cervix. Colposcopic direct biopsy was diagnosed for suspected of AIS, endocervical type.

In accordance with the guidelines of the European Society for Colposcopy and Cervical Pathology (ESCCP) [14] a diagnostic cone biopsy was indicated to define the lesion and to exclude invasive diseases. After proper counselling about the risks and benefits of the procedure during pregnancy, the woman signed her informed consent and underwent a laser conization at 13 weeks plus 1 day in her gestation.

The colposcopic guide laser conization was performed with a Surgilase 40 CO_2_ laser, at a power setting of 40 watt/cm^2^, connected to a micromanipulator mounted on a Zeiss colposcope with a focal spot size of 0.2 mm, under local anaesthesia (cervical injections of 3.0–5.0 mL of a 2% lidocaine). Before and after the procedure, the vitality of the embryo was checked by ultrasound examination. There were no intraoperative or postoperative complications, and there was no need for sticks. Blood loss was negligible. The presurgery length of the cervical canal, measured by ultrasound, was 4.5 cm, while postsurgery length was 3.5 cm, and therefore no cervical cerclage was performed.

After surgery the woman took 341 mg of intramuscular hydroxyprogesterone caproate once a day, every three days for three vials in total, as prophylaxis for uterine hypercontractility, and stayed in bed for 24 hours. The patient was discharged from the hospital on the second day.

The specimen of conization was a truncated cone, 1 cm in height, with a 2 cm larger base and a 0.6 cm shorter base, weighing 3 grams. For the histological analysis, longitudinal sections were taken at regular 2.5 mm intervals across the conization and the whole specimen was included. The submitted tissue was processed. Sections of 5 *μ*m thickness were cut from the formalin-fixed, paraffin embedded tissue blocks. The sections were then stained with haematoxylin and eosin. The pathologic diagnosis was an invasive adenocarcinoma of endocervical type, grade 1 differentiation, with a stromal invasion of 1 mm in depth, and a 3 mm largest superficial extension, close to the SCJ, without lymph-vascular space invasion (LVSI). The section margins of the cone were clear. Clearance from invasive disease was 2 mm both from the margins and the apex of the cone. Definitive FIGO stage was pT1a1 (TNM 7th edition).

After multidisciplinary counselling the patient accepted conization as definitive treatment until the delivery, and a followup every eight weeks during pregnancy with pap smear, colposcopy, and, if indicated, biopsy is planned. A revaluation is planned three months after the delivery to decide on hysterectomy, as required by the European guidelines [14].

Until the delivery the course of pregnancy was normal. Pap smears and colposcopies were negative and no biopsy was performed. No obstetric complication was recorded. The growth of the fetus was regular, and the length of the cervical canal was 4.0 cm at 31 gestational weeks. The patient delivered vaginally at 40 weeks plus 3 days of gestation.

The first stage of labour lasted 155 minutes; the second stage lasted 44 minutes, and a vacuum extractor was applied because of the delayed progression of the presenting part at low pelvic level.

Apgar's Score was 9 in the first minute and 10 at the fifth and tenth minutes. The weight was 3090 grams, and the length was 47 cm. The blood loss was 100 mL.

At 12 and 24 weeks from the delivery the woman underwent pap smear, colposcopy, and cervical curettage. A Hybrid Capture 2 HPV DNA test (HC2-HPV test) was performed at 24 weeks. Colposcopies were satisfactory, the SCJ was visible, and the transformation zone (TZ) was normal. Pap, cervical curettage, and HC2-HPV tests were always negative.

After 24 weeks from delivery, the patient expressed her desire for another child and signed her informed consent to consider the conization performed during pregnancy as definitive treatment, at least up to a next pregnancy. She declared also to be available for followup, planned quarterly with pap smear, colposcopy, cervical curettage, and after a year, HC2-HPV test. At 18 months from the delivery, pap smear, colposcopy, cervical curettage, and HC2-HPV tests were negative.

## 3. Discussion

The microinvasive carcinoma of the uterine cervix may affect women in their reproductive age who deeply desire to become pregnant. To preserve fertility in these patients, a conservative approach has been studied. Conservative surgery may be effective in MISC, but as far as MIAC is concerned there are few studies and there are no definite data that recommend conservative treatment. Adenocarcinoma lesions have a heterogeneous natural history, with a different histological type and different connections with HPV infections. For example, although endocervical type lesions rise close to the SCJ in more than 90% of cases, other histotypes, such as endometrioid or intestinal ones, commonly arise in any place along the cervical canal [15].

Because of multifocal disease and skip lesions, there may be residual or recurrent glandular neoplasia even in case of apparently negative surgical margins. This situation is more dangerous and feared, since follow-up methods are unreliable for endocervical glandular lesions; colposcopy is a blind method for these lesions and data on the reliability of the HPV test in the followup of invasive glandular disease [16] are not enough. Pap smear is not considered to be a reliable method for the followup of glandular lesions, even if data show a good sensitivity of cervical cytology even for MIAC [17]. Another concern may be the difficulty to exactly stage the microinvasive glandular lesions. The lesion is measured from the point of origin in the basal membrane of the epithelium or crypt, but the invasion is not easy to identify, and at this point it may be impossible to determine, even for experienced pathologists [1]. Invasion is more readily identified beyond the normal glandular field; a stromal response of oedema, loose fibrosis, and inflammation often accompany invasion. Despite this, a conservative treatment seems to be possible for endocervical HPV-related histotype adenocarcinomas, arising close to the TZ. All these aspects are critical during the pregnancy. The histological diagnosis of MIAC may be more difficult because of the changes induced by pregnancy hormones on the cervix itself. In the normal endocervical epithelium there is a measurable increase in the length and tortuosity of endocervical crypts; the columnar epithelium becomes multilayered and may form papillary projections. Because of progesteron activity, Arias-Stella reaction is characterized by hypertrophy and vacuolization of glandular epithelial cells, associated with marked nuclear pleomorphism, enlargement, and hyperchromasia [18]. Sometimes decidualisation of the stroma is accompanied by the disruption of the overlying mucosae [18]. All these findings can determine difficulties in defining whether glandular neoplasia is in situ; defining exactly the extension of invasion, if present, may be difficult too. Similarly, colposcopy exams in pregnancy should be performed by an expert colposcopist because of the difficulty to rightly evaluate the modifications in the gestational cervix [19].

Conization has been chosen to find out invasive diseases, but complications, such as bleeding, risk of abortion, premature labour, and/or premature rupture of membranes may arise in pregnancy; the last one has been related to an infection in the cervical canal, especially if this is less than 2.5 cm long [20], and this residual length has been advocated for cervical incompetence [21]. However, conization in pregnancy is appropriated if there is a real suspicion of invasion and to perform a conclusive diagnosis in presence of AIS to find out invasive diseases [14]. Because of these restricted indications, cone biopsy during pregnancy is rare, but when performed, “loop conization” has been advocated [22]. Laser cone biopsy has proved to be safe when performed between 12 and 18 weeks of pregnancy, when the height of the cone is no more than 20 mm [23, 24], while a poor obstetric outcome with an increased frequency of miscarriage and preterm delivery has been described when cone biopsy is performed during the first trimester. Yahata and colleagues performed conization in their series from 16 to 23 gestational weeks [25]. In this study, a CO2 laser cone biopsy at 13 weeks of pregnancy was performed for a completely visible lesion close to the SCJ, suspected of adenocarcinoma in situ, endocervical type. The definitive diagnosis was MIAC, endocervical type, grade 1 with stromal invasion of 1.0 mm in depth, a 3.00 mm largest superficial extension, close to the SCJ, and without lymphovascular space invasion. The section margins of the cone were clear; the residual length of the cervix was more than 3.5 cm and cervical cerclage was not performed. Multidisciplinary counselling was offered after definitive histology, and particular attention was called on the limits of a conservative treatment in MIAC, which is less safe and effective than in MISC. The risk of recurrence, the poor prognosis reported in the literature [26, 27], and the lack of data about conservative treatment in pregnancy were underlined.

Because of the low reliability of follow-up methods, a simple hysterectomy was offered after delivery, but the patient did not consider her reproductive life finished and refused definitive surgery.

At 18 months from delivery, the patient was alive and free of disease but she has not achieved pregnancy. To the best of our knowledge, there are not enough data in the medical literature to classify this choice for adenocarcinoma as safe, although it is now recognized that the histological cell type (squamous or glandular) has no impact on survival for stage I diseases [28].

Yahata and colleagues performed radical demolitive surgery in 3 out of 4 of their patients [25], but the wishes of the woman have been respected. This case is thought to have a low risk of recurrence because of the endocervical type, grade 1 differentiation, and lack of LVSI; indeed, an increased risk of recurrence is correlated with endometrioid and other histological types [29], LVSI [29], and grade 3 differentiation [30]. However, although the pregnancy status per se is not a pejorative factor for the prognosis and the currently available data not concerning pregnancy are in favour of a conservative treatment for stage Ia1 MIAC, there is the risk of a higher possibility of unrecognized residual diseases and underestimated disease extension, specifically in pregnancy.

It is well known that the survival after simple total hysterectomy is the same as after radical surgery [31], and there are data about long-term followup after conservative treatment [9, 29]. Only conization or a simple/radical trachelectomy—eventually associated with lymphadenectomy—may be defined as “conservative treatment,” which is to say a fertility-sparing treatment. We do not agree with authors who include women treated with simple hysterectomy in the group of women treated conservatively because of MIAC [31] in their studies.

## 4. Conclusions

It is important to perform a pap test in the early stages of pregnancy if it has not been already performed in the last three years.

The diagnosis of MIAC during pregnancy is a rare condition which has to be managed by clinicians and pathologists specialized in gynecological oncology, and specifically in cervical pathology. In stage Ia1 disease, endocervical type, with clear margins and without LVSI, cervical conization performed during the second trimester may be considered a definitive and safe treatment, at least up to delivery, after expression of informed consent by the woman. In this case, no obstetric complication has been recorded after cervical conization and, after a followup of 18 months, the patient was alive and free of disease, with negative results as far as pap smear, colposcopy, HPV status, and cervical curettage are concerned. The decision on radical (demolitive surgery) treatment was discussed 24 weeks from the delivery but was postponed because the patient expressed her desire for another child and signed her informed consent to consider the conization performed during pregnancy as definitive treatment.
